# Supplementary material for: Genetic Strain Diversity of Multi-Host RNA Viruses that Infect a Wide Range of Pollinators and Associates is Shaped by Geographic Origins
Source: Viruses. 2020 Mar 24;12(3):358. doi: 10.3390/v12030358 (PMC7150836; doi:10.3390/v12030358)
Supplement: Supplementary file 1 [file viruses-12-00358-s001.zip › Supplementary Materials pollinator RNA virus.pdf]

Supplemental material:

Genetic strain diversity of multi-host RNA viruses that infect a wide range of pollinators and associates is shaped by geographic origins

Jana Dobelmann[1*], Antoine Felden[1] and Philip J. Lester [1]

[1] School of Biological Sciences, Victoria University of Wellington, Wellington, New Zealand; jana.dobelmann@vuw.ac.nz

* Correspondence: jana.dobelmann@vuw.ac.nz

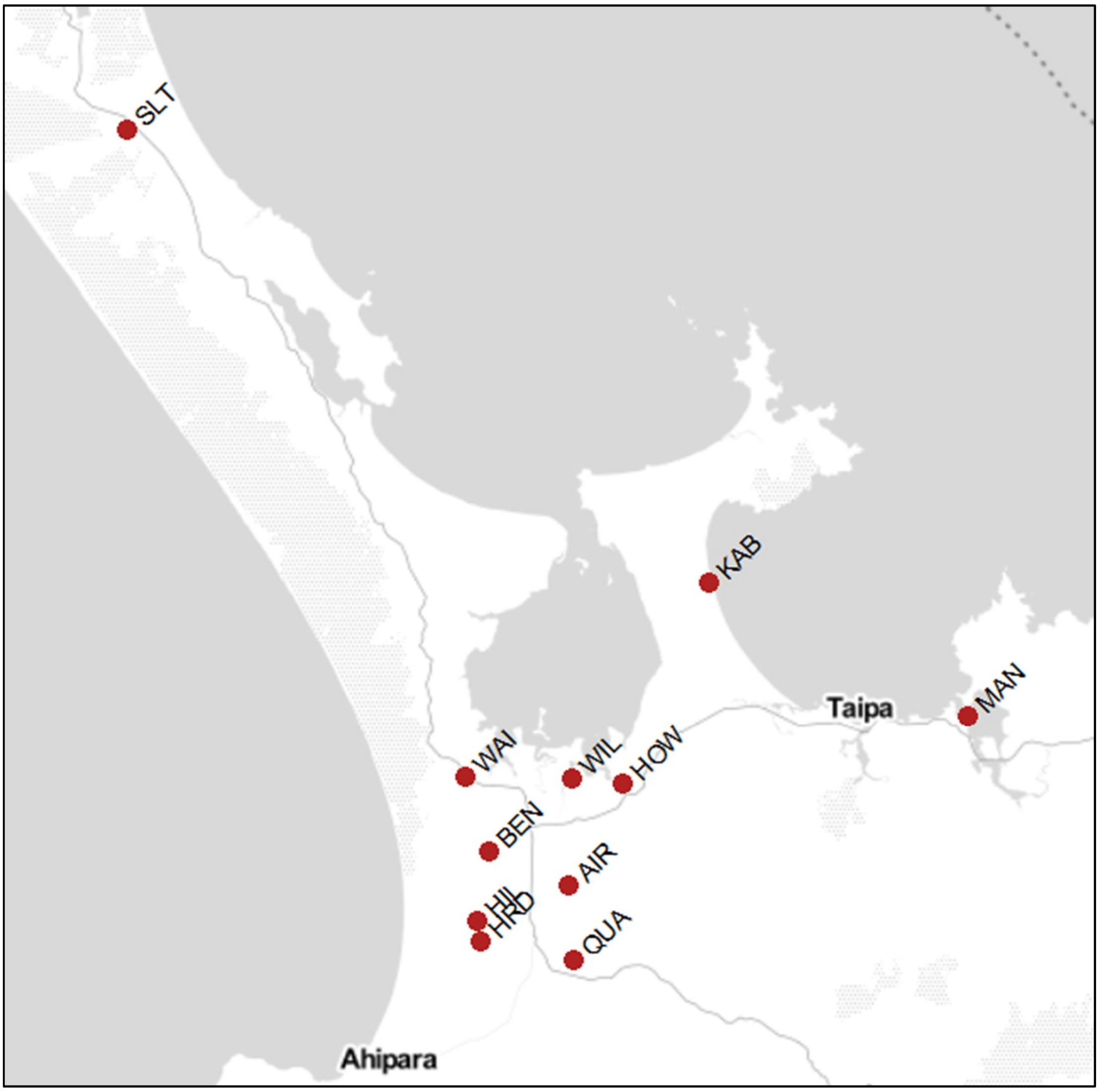

Figure S1: Sampling sites in the far north region near Ahipara, Northland, New Zealand. AIR, BEN, WAI are sampling locations in apiaries; WAI and SLT had Argentine ant nests; all other sites without apiaries or Argentine ant nests nearby. QUA:-35.116650, 173.285114; AIR: -35.077690, 173.282120; BEN: -35.060490, 173.231160; WAI: -35.021770, 173.216260; SLT: -34.681947, 173.000169; KAB: -34.919640,

173.371398; HRD: -35.106948, 173.225998; MAN: -34.989425, 173.536581; HOW: -35.024903, 173.316340; WIL: -35.022270, 173.283750; HIL: -35.096685, 173.223495.

.

Table S1: Primers used for virus detection. Screened viruses are deformed wing virus (DWV), black queen cell virus (BQCV), Kashmir bee virus (KBV), Linepithema humile bunya/like virus 1 (LhuBLV1) and Moku virus (MKV). Primers were used in presence/sbsemce PCR and sequencing (presence/absence) or in strand specific RT-PCR ((-) strand or (+) strand. Forward primer (F) and reverse primer (R), tagged primer (tag-F/tag-R) are given in 3' to 5' direction, with the length of the PCR product in base pairs (pb) and the annealing temperature used in PCR reactions (anneal. T).

Virus	Target gene	Method	Primer sequence	[bp]	T [°C]	Ref.
DWV	RdRP	+/-	F: TCCATCAGGTTCTCCAATAACGGA<br>R: CCACCCAAATGCTAACTCTAAGCG	451	55	[1]
		Repl.	tag-F: agcctgcgcaccgtggTCCATCAGGTTCTCCAATAACGGA			
BQCV	Capsid/ 3'UTR	+/-	F:TGGTCAGCTCCCACTACCTTAAAC<br>R:GCAACAAGAAGAAACGTAAACCAC	700	56	[2]<br>[3]
KBV	Capsid	+/-	F: TGTTTGTGGCAATCCAGCTA<br>R: TACGTCTTCTGCCCATTTCC	625	54	
		Repl.	Tag-F: agcctgcgcaccgtggTGTTTGTGGCAATCCAGCTA			
LhuBLV1	RdPR	+/-	F:GTCACAGAGGAGCAGATACCA<br>R:GCCCATAAACCTTGCTCTGC	89	54	[4]
		Repl.	Tag-R: agcctgcgcaccgtggGCCCATAAACCTTGCTCTGC			
MKV	Polyprot.	+/-	F: CTACAACGCACGCGAGTAGA<br>R:CCTTTCAAAGCAACGCTACC	102	55	[5]
		Repl.	Tag-F: agcctgcgcaccgtggCTACAACGCACGCGAGTAGA			

Table S2: Sampling location of additional Argentine ant samples used for phylogenetic analyses from Felden et al. 2019 [6] .

Country -Location	Latitude	Longitude	Collection date
**France**			
**-Toulouse**	43.5593	1.4723	August 2016
**Argentina**			
**-Otamendi**	-34.2224	-58.8961	January 2015
**New Zealand**			
**-Oakura**	-39.1195	173.9397	February 2016
**-Paraparaumu**	-40.963439	174.973731	February 2016

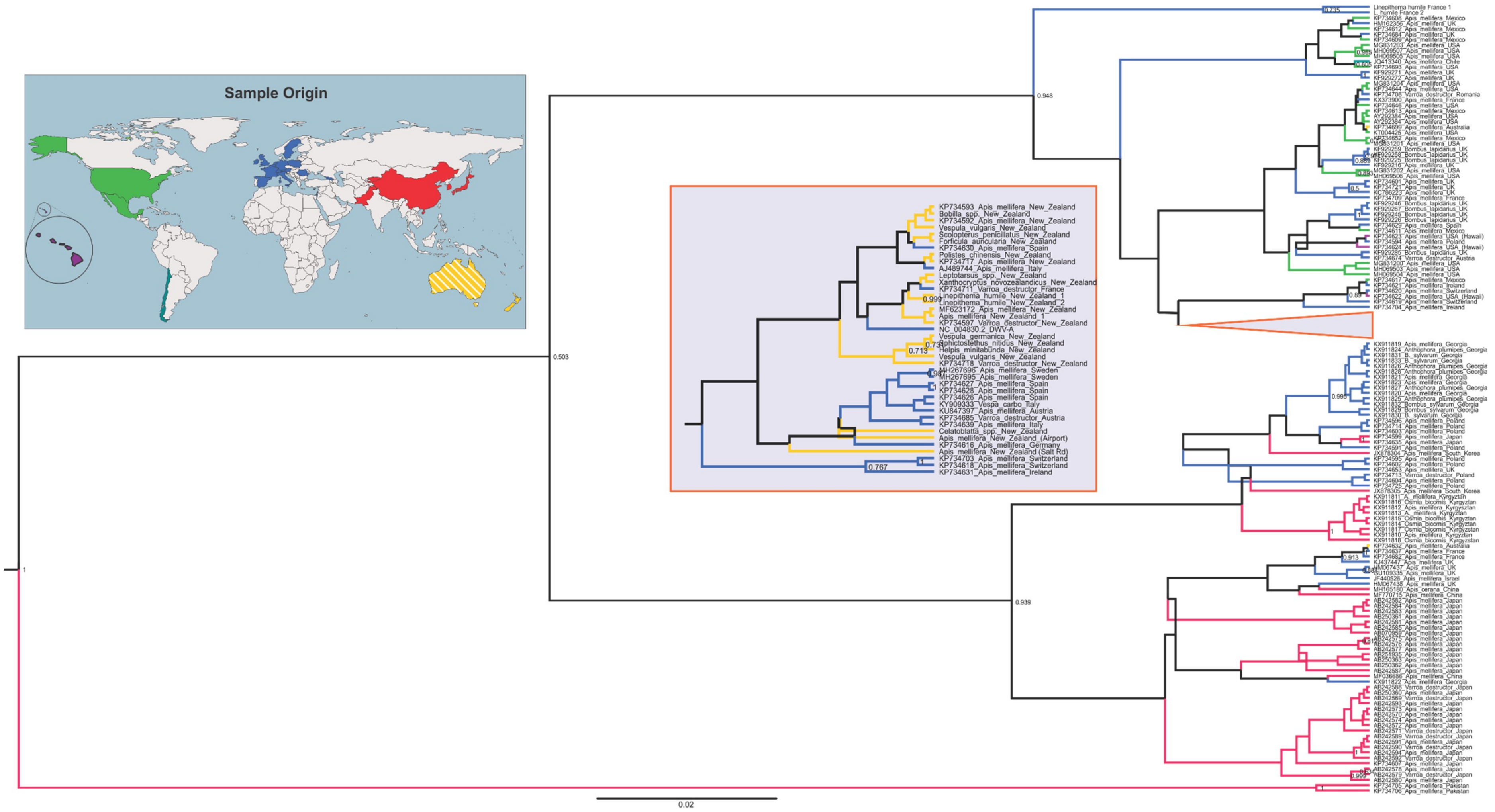

Figure S2: Non-dated maximum clade credibility tree for the RdRp fragment (440 bp) of deformed wing virus (DWV) without tip dates. Grey insert with orange frame shows collapsed part of the tree that among others includes the 19 samples from New Zealand from this study. Species name, country of origin and if applicable GenBank accession number are given in the branch label. The branches are coloured according to the lineages' inferred geographic origin as shown on the world map. Posterior support > 0.5 is given and the scale bar shows substitutions.

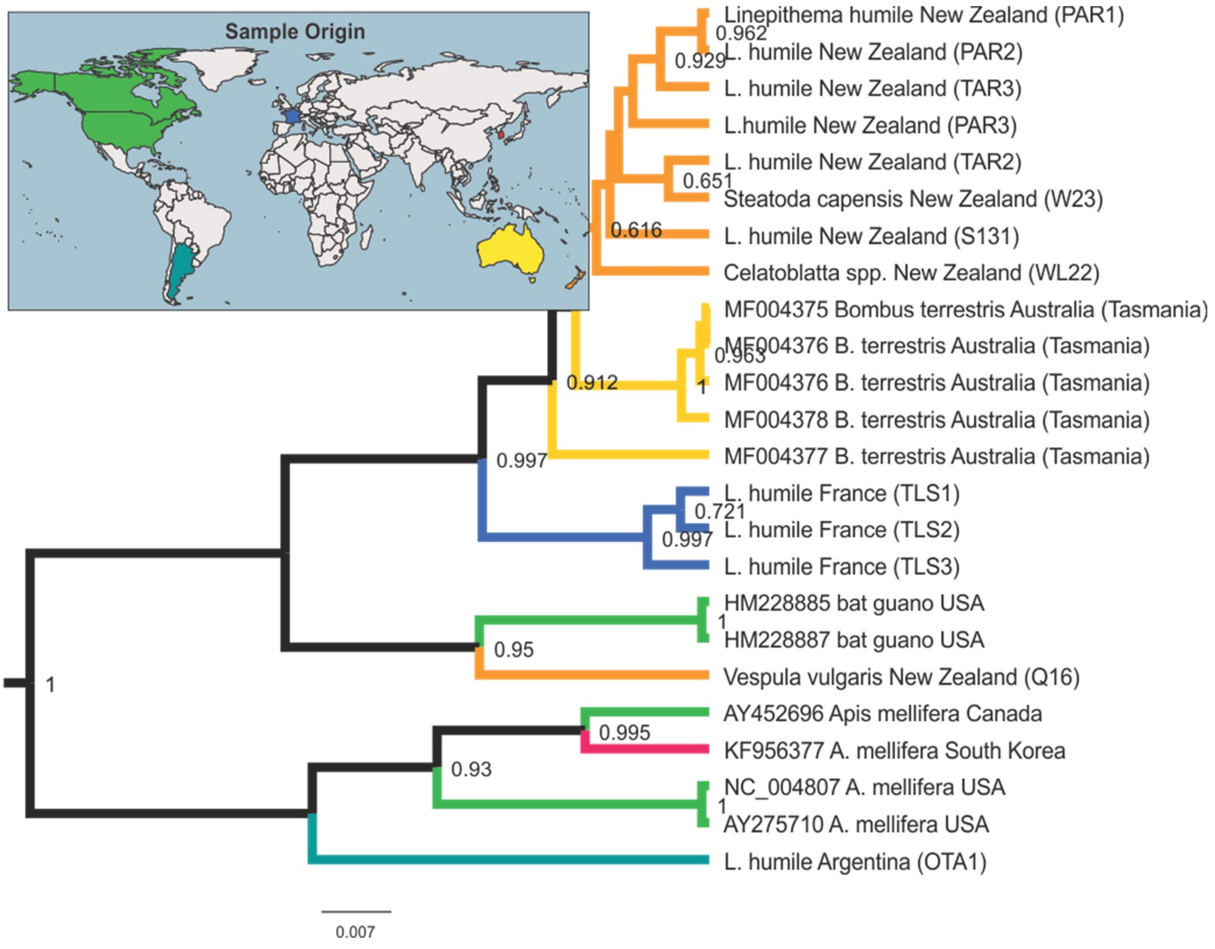

Figure S3: Non-dated maximum clade credibility tree for the vp3 fragment (360 bp) of Kashmir bee virus (KBV) without tip dates. Species name, country of origin and if applicable GenBank accession number are given in the branch label. The branches are coloured according to the lineages' inferred geographic origin as shown on the world map. Posterior support > 0.5 is given and the scale bar shows substitutions.

Table S3: Trait-tip association of non-dated Deformed wing virus (DWV) phylogeny, as calculated with 100 random trees (replicates) in BaTS [7]. Low parsimony score (PS) represents fewer state changes on the tree and stronger trait-tip association. Maximum clade (MC) scores show the maximum size of a clade for a trait state, high MC is positively correlated with trait-tip association.

Statistic	Observed /expected	n	observed mean	lower 95% CI	upper 95% CI	null mean	lower 95% CI	upper 95% CI	p-value
Association Index (AI)									
Geographic origin	0.26		3.82	2.81	4.84	14.80	13.86	15.68	**< 0.01**
Host species	0.59		5.82	4.72	6.89	9.87	9.22	10.44	**< 0.01**
Parsimony Score (PS)									
Geographic origin	0.36		31.43	28.00	35.00	88.49	85.33	91.86	**< 0.01**
Host species	0.72		37.07	34.00	40.00	51.26	50.05	52.07	**< 0.01**
Maximum Clade (MC) scores									
Apis		126	14.72	9.00	24.00	7.48	5.93	9.63	0.02
Varroa destructor		13	1.67	1.00	3.00	1.20	1.02	1.49	0.03
Associate		6	1.31	1.00	2.00	1.05	1.00	1.16	1
Non-*Apis* bee		23	4.23	4.00	6.00	1.66	1.31	2.05	**< 0.01**
Vespidae		5	1.07	1.00	2.00	1.04	1.00	1.15	1
Formicidae		4	2.00	2.00	2.00	1.01	1.00	1.05	**< 0.01**
Other Hymenoptera		2	1.00	1.00	1.00	1.01	1.00	1.01	1
Asia		50	21.52	13.00	34	2.58	2.23	3.28	**< 0.01**
Europe		76	14.79	14.00	19.00	3.63	3.03	4.44	**< 0.01**
North America		24	3.80	3.00	6.00	1.70	1.31	2.08	**< 0.01**
Oceania		25	6.09	4.00	10.00	1.85	1.49	2.50	**< 0.01**
South America		1	1.00	1.00	1.00	1.00	1.00	1.00	1
Hawaii		3	1.05	1.00	2.00	1.03	1.00	1.08	1

Table S4: Trait-tip association of non-dated Kashmir bee virus (KBV) phylogeny, as calculated with 100 random trees (replicates) in BaTS [7]. Low parsimony score (PS) represents fewer state changes on the tree and stronger trait-tip association. Maximum clade (MC) scores show the maximum size of a clade for a trait state, high MC is positively correlated with trait-tip association.

Statistic	Observed /expected	n	observed mean	lower 95% CI	upper 95% CI	null mean	lower 95% CI	upper 95% CI	p-value
Association Index (*AI*)									
Geographic origin	0.18		0.40	0.33	0.62	2.22	1.74	2.64	**< 0.01**
Host species	0.20		0.42	0.13	0.66	2.08	1.47	2.56	**< 0.01**
Parsimony Score (*PS*)									
Geographic origin	0.46		6.26	6.00	7.00	13.55	12.01	14.90	**< 0.01**
Host species	0.53		6.61	6.00	7.00	12.43	10.78	13.79	**< 0.01**
Maximum Clade (*MC*) *scores*									
Apis mellifera		6	3.86	2.00	4.00	1.37	1.00	2.17	**< 0.01**
other		5	2.95	2.00	3.00	1.26	1.00	2.00	**< 0.01**
Linepithema humile		10	3.50	3.00	4.00	2.01	1.10	3.06	0.09
Bombus		3	2.95	3.00	3.00	1.09	1.00	1.72	**< 0.01**
North America		4	2.00	2.00	2.00	1.34	1.00	2.00	0.15
New Zealand		9	7.12	4.00	8.00	1.99	1.05	3.10	**< 0.01**
South America		1	1.00	1.00	1.00	1.00	1.00	1.00	1.00
Europe		3	3.00	3.00	3.00	1.10	1.00	2.00	**< 0.01**
Asia		1	1.00	1.00	1.00	1.00	1.00	1.00	1.00
Australia		5	4.35	4.00	5.00	1.27	1.00	2.00	**< 0.01**

References

1. Yue, C.; Genersch, E. RT-PCR analysis of *deformed wing virus* in honeybees (*Apis mellifera*) and mites (*Varroa destructor*). *J Gen Virol* **2005**, *86*, 3419-3424, doi:10.1099/vir.0.81401-0.
2. Singh, R.; Levitt, A.L.; Rajotte, E.G.; Holmes, E.C.; Ostiguy, N.; Vanengelsdorp, D.; Lipkin, W.I.; Depamphilis, C.W.; Toth, A.L.; Cox-Foster, D.L. RNA viruses in hymenopteran pollinators: Evidence of inter-Taxa virus transmission via pollen and potential impact on non-*Apis* hymenopteran species. *PLoS One* **2010**, *5*, e14357, doi:10.1371/journal.pone.0014357.
3. Benjeddou, M.; Leat, N.; Allsopp, M.; Davison, S. Detection of *acute bee paralysis virus* and *black queen cell virus* from honeybees by reverse transcriptase PCR. *Appl Environ Microbiol* **2001**, *67*, 2384-2387, doi:10.1128/AEM.67.5.2384-2387.2001.
4. Viljakainen, L.; Holmberg, I.; Abril, S.; Jurvansuu, J. Viruses of invasive Argentine ants from the European Main supercolony: Characterization, interactions and evolution. *J Gen Virol* **2018**, *99*, 1129-1140, doi:10.1099/jgv.0.001104.
5. Garigliany, M.; Taminiau, B.; El Agrebi, N.; Cadar, D.; Gilliaux, G.; Hue, M.; Desmecht, D.; Daube, G.; Linden, A.; Farnir, F., et al. Moku Virus in invasive Asian hornets, Belgium, 2016. *Emerg Infect Dis* **2017**, *23*, 2109-2112, doi:10.3201/eid2312.171080.
6. Felden, A.; Paris, C.; Chapple, D.G.; Suarez, A.V.; Tsutsui, N.D.; Lester, P.J.; Gruber, M.A. Native and introduced Argentine ant populations are characterised by distinct transcriptomic signatures associated with behaviour and immunity. *NeoBiota* **2019**, *49*, 105-126, doi:10.3897/neobiota.49.36086.
7. Parker, J.; Rambaut, A.; Pybus, O.G. Correlating viral phenotypes with phylogeny: Accounting for phylogenetic uncertainty. *Infect Genet Evol* **2008**, *8*, 239-246, doi:10.1016/j.meegid.2007.08.001.
